# Sage Tea for Hiccough

**Published:** 1895-04

**Authors:** N. F. Howard

**Affiliations:** Dahlonega, Ga.


### *CORRESPONDENCE.*

---

### SAGE TEA FOR HICCOUGH.

DAHLONEGA, GA., February 10, 1895.

*Atlanta Medical and Surgical Jonrnal:*

In 1868, Mr. H., of Amaria, this county, merchant, aged fifty years, had an attack of typhoid fever, and at the end of thirty days, when very much prostrated, was taken with hiccough, which continued with severity for a day or two without yielding to any of the usual remedies for this trouble at our command. We then decided to put sage tea on trial. A pint of strong sage tea was prepared, and Mr. H. drank the most of it as hot as he could comfortably swallow it. To our surprise the hiccough was immediately arrested, and the patient slept well the most of that night. In about eight hours the trouble returned. A cup of hot sage tea, however, again put a stop to the spell. The paroxysms continued to recur at intervals, but milder, and the tea invariably relieving the paroxysms, until after a few days the hiccough entirely disappeared. The patient improved, and in due time recovered, and is yet living.

Case 2.—Mr. J. D., farmer and laborer, aged forty-six years. This is rather a unique case. Mr. D. informs me that, from a child up to the time I prescribed for him, whenever he became excited, fatigued, worked in the sun, or drank intoxicants, he would have an attack of hiccough and become very sick for a time. In 1888, I met him on the streets of Dahlonega, just recovering from a "big drunk," as he called it, and suffering most painfully with the hiccough. I prescribed a pint of hot sage tea, and told him it would "cure" him of his drunk and hiccough. He at once prepared it, sweetened it with "sorghum" and drank it, and was immediately relieved. Mr. D. told me, the other day, that he had not had another spell of hiccough since he drank the sage tea, nearly seven years ago, yet had often been under the influence of intoxicants.

Case 3.—Mrs. D., aged sixty-five years, suffered with cancer for some years. Before her death, September, 1890, for weeks had hiccough; and when all other remedies lost their influence, sage tea never failed to afford relief for a time, as long as Mrs. D. lived.

These three cases are selected and reported as being, perhaps, the most severe of any treated by me; but in a number of other cases when the sage tea was used, the result was the same as in these cases. N. F. HOWARD, M.D.

---

### LABOR AND HEART-DISEASE.

Tarnier (*Journal des Sages-Femmes,* January 16, 1894) notes that in heart-disease all great and sudden efforts put the patient in peril, and labor is no exception to the rule. Running up-stairs, racing to catch an omnibus or train, and sexual intercourse may all cause fatal syncope. The danger of labor is not special in this sense; it is dangerous in heart-disease simply because it involves much effort. Tarnier induced premature labor in lady who was subject to advanced heart-disease. Notwithstanding all precautions, she became moribund in the course of the labor. Directly she died, he turned and delivered a live child, which survived. A woman was brought into Tarnier's wards in January, 1894, in labor, with advanced heart-disease and asystolism; she was apparently dying. Immediately about 300 grammes of blood were withdrawn, and the symptoms of suffocation diminished. The patient grew calmer. As it was extremely advisable to bring on labor quickly, as the forceps is apt to fatigue the patient, and as, in particular, the child was dead, the basiotribe was applied and delivery effected. A few days later the mother was doing very well.—*British Medical Journal.*

